# Effect of direct endovascular treatment versus standard bridging therapy in large artery anterior circulation stroke (DEVT): 18-month follow-up of a randomized controlled trial

**DOI:** 10.1186/s12883-023-03111-y

**Published:** 2023-02-27

**Authors:** Zhongfan Ruan, Xiaojun Luo, Yongkang Liu, Fengli Li, Jincheng Liu, Wenjie Zi

**Affiliations:** 1 Department of Neurology, The First Affiliated Hospital, Hengyang Medical School, University of South China, Hengyang, Hunan China; 2 Department of Cerebrovascular Diseases, Guangyuan Central Hospital, Guangyuan, Sichuan China; 3 Department of Cerebrovascular Diseases, Zhuhai Hospital Affiliated With Jinan University (Zhuhai People’s Hospital), Zhuhai, Guangdong China; 4 Department of Neurology, Xinqiao Hospital and the second affiliated hospital of Army Medical University (Third Military Medical University), No. 183 Xinqiao Main Street, Shapingba District, Chongqing, 400037 China; 5 Department of Neurology, Xiangyang Hospital of Traditional Chinese Medicine, Xiangyang, Hubei China

**Keywords:** Endovascular treatment, Bridging therapy, Long-term outcome

## Abstract

**Background:**

Two trials in Chinese population showed that endovascular treatment (EVT) alone was noninferior to alteplase follow by EVT at 90 days. However, results of long-term clinical outcomes remain unknown. We reported the results of prespecified 18-month analysis of the DEVT trail.

**Materials and methods:**

We assessed clinical outcomes 18 months after patients were randomly assigned to receive EVT alone or bridging therapy for acute ischemic stroke (AIS). The primary outcome was the proportion of functional independence [modified Rankin scale (mRS), 0–2] at 18 months. Secondary outcomes included all-cause mortality and the quality of life at 18 months as measured by means of a health utility index according to the European Quality of Life 5-Dimension 5-level scale (EQ-5D-5L). Kaplan-Meier event curves were used to investigate the risk of mortality in participants with EVT alone or bridging therapy.

**Results:**

Among 234 patients (EVT alone, *n* = 116; bridging therapy, *n* = 118) in the DEVT trial, only 231 (98.7%) patients were extended follow-up to 18 months. A total of 60 (51.7%) patients in the EVT alone achieved functional independence vs 56 (47.5%) patients in the bridging therapy (difference, 4.3%; 1-sided 97.5% CI, − 8.4% to ∞, P for noninferiority =0.014). No significant between-group difference was detected in EQ-5D-5L score (0.81 vs 0.73; difference, 0; 95% CI, 0 to 0.005). The cumulative mortality was 27.6% in the EVT alone and 28.8% in the bridging therapy.

**Conclusion:**

At 18 months follow-up, EVT alone was noninferior to bridging therapy regarding favorable functional outcome in patients with AIS.

**Trial registration:**

Trial was registered on Chinese Clinical Trial Registry (ChiCTR-IOR-17013568) on 27/11/2017.

**Supplementary Information:**

The online version contains supplementary material available at 10.1186/s12883-023-03111-y.

## Introduction

Randomized studies have established endovascular treatment (EVT) as a standard of care for acute ischemic stroke (AIS) due to large vessel occlusion (LVO) in the anterior circulation [1–7]. However, it remains controversial whether intravenous thrombolysis (IVT) prior to EVT is superior to direct EVT. Several clinical trials didn’t reach the prespecified statistical threshold for noninferiority for functional independence, and no significant differences were detected in functional independence at 90 days [8–10]. However, the results of the Multicenter Randomized Clinical Trial of Direct Endovascular Treatment Versus Standard Bridging Therapy in Large Artery Anterior Circulation Stroke (DEVT) demonstrated that EVT alone is noninferior to alteplase followed by EVT in terms of functional independence at 90 days [11]. This study finding was consistent with that reported recently in the Direct Intraarterial Thrombectomy in Order to Revascularize Acute Ischemic Efficiently in Chinese Tertiary Hospitals (DIRECT-MT) trial [12]. However, whether this noninferiority is sustained during a long-term follow-up was lacking, particularly in developing countries which had a higher stroke burden, but more limited health care resources. In addition, the information of long-term clinical outcomes may be useful for routine clinical practice. Therefore, in the current paper, we report the results of clinical follow-up at 18 months after randomization among patients in the DEVT trial.

## Methods

### Trial design and oversight

The DEVT was a multicenter clinical trial with randomized treatment allocation, open-label treatment, and a blinded endpoint evaluation. The interventions were either EVT alone or intravenous alteplase plus EVT in patients with proximal anterior circulation occlusions treated within 4.5 hours of onset. All patients were admitted to acute stroke units, or intensive care units if needed, and treated following national and international guidelines. Detailed information about the main trial, including the treatments, blinding, statistical analysis, and determination of sample size, were described in the protocol and statistical analysis plan (SAP) of the DEVT [9, 11, 12].

Trial investigators at the coordinating center collected the data for this extended follow-up trial. Members of the DEVT executive committee designed the extended follow-up trial, analyzed the data, prepared the manuscript, and made the decision to submit the manuscript for publication. The authors vouch for the accuracy and completeness of the available data from the extended follow-up trial and for the fidelity of the trial to the protocol. The study protocol was approved by medical ethics committee of the Second Affiliated Hospital of the Army Medical University and all participating centers. Written informed consent was obtained from the patient or patient’s representative, as required by national and local guidelines.

### Patients

Patients aged 18 years or older with acute ischemic strokes caused by anterior proximal artery occlusions (intracranial internal carotid artery or M1 segment of the middle cerebral artery) who could be treated with intravenous alteplase treatment within 4.5 hours of stroke onset were eligible for inclusion in the DEVT. Time of stroke onset was defined as when the patient was last known to be well. Detailed information about the inclusion and exclusion criteria were described in the protocol of the DEVT trial [13].

Two trial investigators, blinded to treatment allocation, centrally assessed functional outcomes using the modified Rankin scale (mRS) scores and recorded the occurrence of medical events between follow-ups on the basis of video or voice recordings taken at the outpatient clinic, during a telephone or video call, or by the patient’s family. If video or voice recordings were unavailable, mRS scores were determined by trained interviewers who were unaware of initial information and was based on a standardized interview protocol, supplemented by medical record review. Disagreements were resolved by consensus. The patient or his or her primary caregiver were also be invited to complete the European Quality of Life 5-Dimension 5-level scale (EQ-5D-5L) questionnaire to assess quality of life [14].

### Outcome measures

The primary outcome was the proportion of patients achieving functional independence (mRS 0–2) at 18 months. The mRS is an ordinal scale that ranges from 0 (no symptoms) to 6 (death) [15]. Secondary outcome measures included:(1) All-cause mortality within 18 months of follow-up and mortality between 3 and 18 months in patients alive at the 3-month follow-up; (2) Distribution of 18-month mRS scores: Improvement based on the classical dichotomization of the 18-month mRS including: mRS 0–1 (excellent outcome) versus 2–6 and mRS 0–3 (favorable outcome) versus 4–6; (3) First-ever major vascular events between 3 and 18 months of follow-up; (4) Quality of life at 18 months, using the EQ-5D-5L questionnaire. The EQ-5D-5L consists of a descriptive system that assesses five dimensions of quality of life: mobility, self-care, usual activities, pain or discomfort, and anxiety or depression.

### Definitions and assessment of major vascular events

For this study, major vascular events included fatal or nonfatal cardiac events, fatal or nonfatal strokes, and fatal or nonfatal major peripheral arterial or thromboembolic events. Cardiac events included myocardial infarction, resuscitation after cardiac arrest, and hospitalization for unstable angina or cardiac insufficiency. Major peripheral events included all events not related to coronary arterial disease leading to hospitalization or revascularization (e.g., new or worsening of claudication leading to revascularization). Major thromboembolic events included pulmonary emboli or cerebral venous thromboses.

We defined a recurrent stroke as a stroke which (1) there was clinical evidence of the sudden onset of a new focal neurological deficit with no apparent cause other than a vascular origin (i.e., the deficit could not be ascribed to an intercurrent acute illness, epileptic seizure, or toxic effect) occurring at any time after the index stroke or (2) clinical evidence of the sudden onset of an exacerbation of a previous focal neurological deficit with no apparent cause other than a vascular origin [16, 17].

Reported events were confirmed by contacting treating physicians, hospitals, and/or general practitioners. All events were centrally reviewed by two investigators who are blinded to treatment allocation.

### Statistical analysis

The main analysis at 18 months was identical to that at 3 months. The primary effective variable was the proportion of patients in each group with an mRS score of 0–2 at 18 months. The primary effective analysis involved the difference between the bridging therapy group (control) and the EVT alone group (test). A binomial comparison was used to test the one-sided null hypothesis that the difference in proportions was less than or equal to − 0.10 (H0: P_test_ - P_control_ ≤ − 0.10) versus the alternative (H1: P_test_, P_control_ > − 0.10), where P_control_ and P_test_ were the proportions of functional independence in the bridging-therapy group and primary thrombectomy group, respectively, which was equivalent to evaluating whether the lower boundary of the two-sided 95% confidence interval for the difference was above − 0.10. The effect variable of this endpoint was analyzed using a logistic regression model with the following terms included: age, baseline NIHSS score, baseline ASPECTS, stroke onset-to-randomization time, and occlusion site. The same imputation for missing values as the one applied in the primary endpoint analysis conducted at 3 months, was applied for the 18 months’ analysis. For subjects missing data for 18-month follow-up, missing values were imputed by assuming the missing modified Rankin scale score at 18-month to be unfavorable. If the patient was known to be alive, we imputed a score of 5. Otherwise, we imputed a score of 6. The analysis based on the per-protocol population was considered supportive.

The treatment effect on dichotomized scores of the modified Rankin scale at 18 months (0 or 1 vs 2 to 6 and 0 to 3 vs 4 to 6) were analyzed with the use of logistic regression, with the odds ratio as the effect variable. An adjusted common odds ratio was applied for a better distribution of outcomes on the modified Rankin scale. All-cause mortality was assessed by means of the Kaplan–Meier method. For the analysis of quality of life, a utility value for each observed EQ-5D-5L health status profile was calculated with the use of an existing algorithm on the basis of valuations elicited by time trade-off techniques applied to the general Chinese population. An unstandardized regression parameter beta was estimated with the use of a multivariable linear regression model and represented the difference between the two treatment groups in the health utility score.

As in the original trial, all effect variables were adjusted for potential imbalances between the two groups in the following prognostic variables at baseline: age, baseline NIHSS score, baseline ASPECTS score, onset to randomization time, and occlusion site. To assess the difference between the two treatment groups in the occurrence of long-term major vascular events, a rate ratio was calculated on the basis of person-years at risk. For subjects missing data for long-term major vascular events, analysis was performed with the use of a regression-based multiple imputation.

Estimates of treatment effects were presented with 95% confidence intervals, unless specified otherwise. A two-tailed *P*-value of < 0.05 was considered significant for all measures. All analyses were based on the intention-to-treat principle. Analyses of the efficacy parameters were adjusted according to the SAP of the main trial, and the results of the unadjusted analysis were also be provided. The analysis was performed after the last randomized patient has reached 18-month follow-up, all data have been validated, the database has been cleaned, and after approval of the SAP by the executive committee. Statistical analyses were performed using SAS version 9.4. Treatment-effect modification was evaluated in the same prespecified subgroups of patients as in the original trial (see the Supplementary Appendix).

## Results

### Trial population

**Fig. 1:**
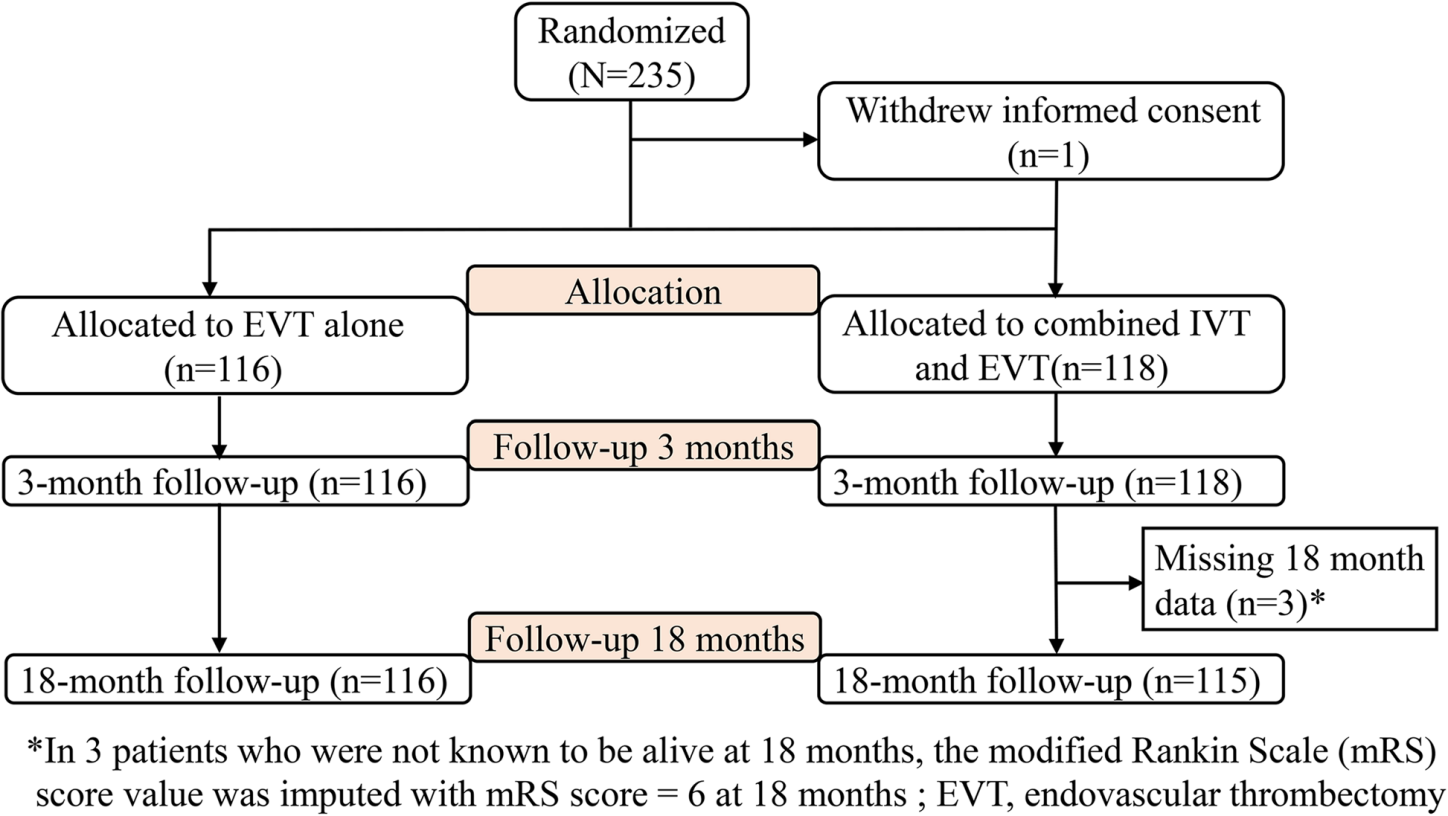
Flow Diagram of the Study

From May 20, 2018 to May 2, 2020, 234 patients were randomly assigned to bridging therapy group (*n* = 118) or EVT alone group (*n* = 116; Fig. 1) in the original trial.

Baseline characteristics were generally similar between the two groups (Table 1). Median age was 70 (60–77) in the EVT alone group and 70 (60–78) in the bridging therapy group. Among all patients, the lower baseline ASPECTS (ASPECTS < 8) were 50 (43.1%) in the EVT alone group and 52 (44.1%) in the bridging therapy group, and the higher NIHSS scores were 59 (50.9%) in the EVT alone group and 65 (55.1%) in the bridging therapy group. The baseline characteristics were consistent with the primary analysis (eTable 1 in Supplement).

**Table 1:** Baseline Characteristics and Workflow Measures

	EVT alone group (*n* = 116)	Bridging therapy group (*n* = 118)
**Demographic characteristics**		
Age,median (IQR), y	70 (60–77)	70 (60–78)
Male sex no.%	66 (56.9)	66 (55.9)
Medical history		
Hypertension	69 (59.5)	74 (62.7)
Atrial fibrillation	62 (53.4)	62 (52.5)
Smoking	28 (24.1)	29 (24.6)
Diabetes	25 (21.6)	20 (16.9)
Hyperlipidemia	18 (15.5)	22 (18.6)
Coronary heart disease	30 (25.9)	19 (16.1)
Clinical characteristics		
Prestroke mRS score		
0	110 (94.8)	107 (90.7)
1	6 (5.2)	11 (9.3)
Stroke etiology		
Cardioembolism	65 (56.0)	69 (58.5)
Large artery atherosclerosis	32 (27.6)	28 (23.7)
Unknown	15 (12.9)	20 (16.9)
Other	4 (3.4)	1 (0.8)
**Imaging characteristics**		
Baseline ASPECTS < 8	50 (43.1)	52 (44.1)
Location of intracranial occlusion		
Intracranial internal carotid artery	18 (15.5)	17 (14.4)
M1 middle cerebra lartery segment	95 (81.9)	99 (83.9)
M2 middle cerebral artery segment	3 (2.6)	2 (1.7)
Baseline NIHSS score ≥ 16	59 (50.9)	65 (55.1)
**Workflow times**		
Time from stroke onset to randomization		
0-3 h	68 (58.6)	68 (57.6)
3-6 h	48 (41.4)	50 (42.4)
Arrival to intravenous alteplase, median (IQR), min	NA	61 (49–81)
Arrival to arterial puncture, median (IQR), min	101 (80–135)	105 (80–132)
Onset to puncture, median (IQR), min	200 (155–247)	210 (179–255)

*Abbreviations*: *ASPECTS* Alberta Stroke Program Early Computed Tomography Score, *IQR* Interquartile range, *EVT* Endovascular treatment, *IVT* Intravenous thrombolysis, *NIHSS* National Institutes of Health Stroke Scale, *NA* Not applicable

### Primary outcome

Data for the primary outcome (mRS score) at 3 months were available for all patients. At 18 months, the data for this outcome variable were available for 231 (98.7%) of 234 patients. All of the 3 patients with missing outcomes at 18 months belong to the bridging therapy group, and the patients were not known to be alive and the mRS score of 6 was imputed.

A total of 60 (51.7%) patients in the EVT alone group achieved functional independence vs 56 (47.5%) patients in the bridging therapy group (difference, 4.3%; 1-sided 97.5% CI, − 8.4%–∞; Table 2). The lower boundary of the CI of − 8.4% was greater than the prespecified noninferiority margin of − 10%. The per-protocol analysis indicated that results were consistent with the primary analysis (eTable 2 in Supplement).

**Table 2:** Modified Rankin Scale Score at 90 Days and Secondary Outcomes

	EVT alone group (*n* = 116)	Bridging therapy group (*n* = 118)	Unadjusted difference (95% CI)	Unadjusted OR (95% CI)	Adjusted OR (95% CI)
Primary efficacy outcome					
Functional independence	60 (51.7)	56 (47.5)	4.3 (−8.4–ꝏ)	1.19 (0.71–1.98)	1.21 (0.68–2.15)
Secondary efficacy outcome					
Excellent outcome	45 (38.8)	46 (39.0)	0.2 (−12.1–12.5)	0.99 (0.59–1.68)	0.96 (0.53–1.74)
Disability level, median (IQR), mRS score	2 (1–6)	3 (1–6)	0 (0–0)	0.96 (0.61–1.51)	0.96 (0.60–1.54)
EQ-5D-5L score, median (IQR)	0.81 (0.00–1.00)	0.73 (0.00–1.00)	0 (0–0.005)	0.01 (−0.11–0.13)	0.004 (−0.01–0.11)

*Abbreviations*: *mRS* Modified Rankin Scale Score, *EQ-5D-5L* European Quality of Life 5-Dimensions 5-Level questionnaire, *IQR* Interquartile range, *EVT* Endovascular treatment, *IVT* Intravenous thrombolysis, *OR* Odds ratio, *CI* Confidence interval

### Secondary outcomes

#### Shift analysis and dichotomized scores on the modified Rankin scale

**Fig. 2:**
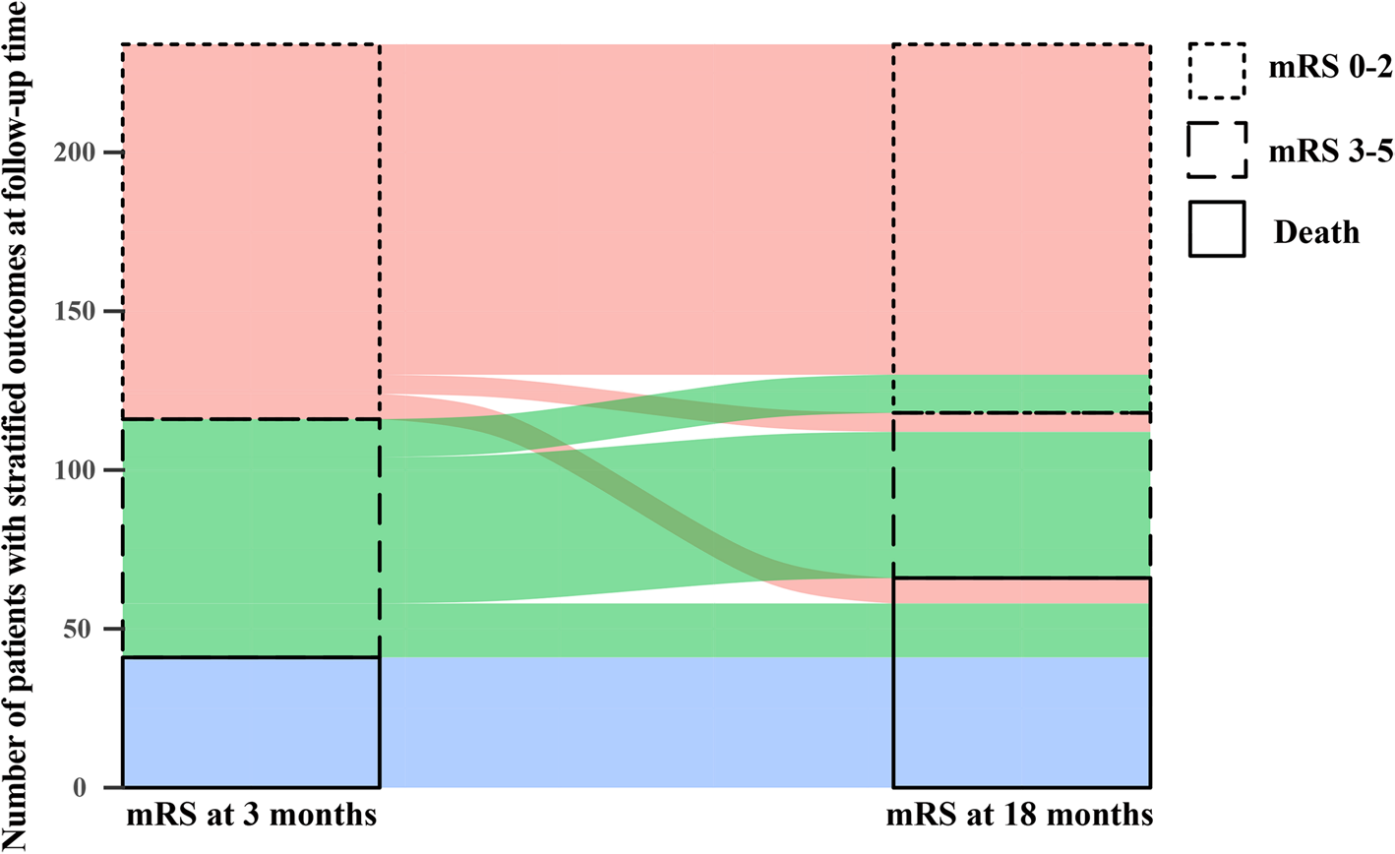
Transformation of outcomes during the follow-up time

The median mRS score was 2 (1–6) in the EVT alone group and 3 (1–6) in the bridging therapy group at follow-up 18 months. Ordinal regression analysis indicated that EVT alone couldn’t increase the functional improvement (adjusted common odds ratio [cOR] 0.96 [95 CI 0.60–1.54]). The number of patients with an excellent outcome (i.e., mRS score 0–1) in the EVT alone group were similar to patients in the bridging therapy group (38.8% vs. 39.0%; adjusted OR, 0.96 [95% CI, 0.53–1.74]) (Table 2). The alluvial diagram, to explore the transformation of outcomes during the follow-up time, suggested that the proportions of functional independence and mortality were increased (Fig. 2). Among patients with a higher quality of life, as assessed with the EQ-5D-5L instrument, no significant difference between the treatment groups was observed (mean health utility score, 0.81 vs 0.73, adjusted β Coefficient, 0.004; 95% CI, − 0.01–0.11) (Table 2). The per-protocol analysis indicated that results were consistent with the primary analysis (eTable 2 in Supplement).

#### All-cause mortality

**Fig. 3:**
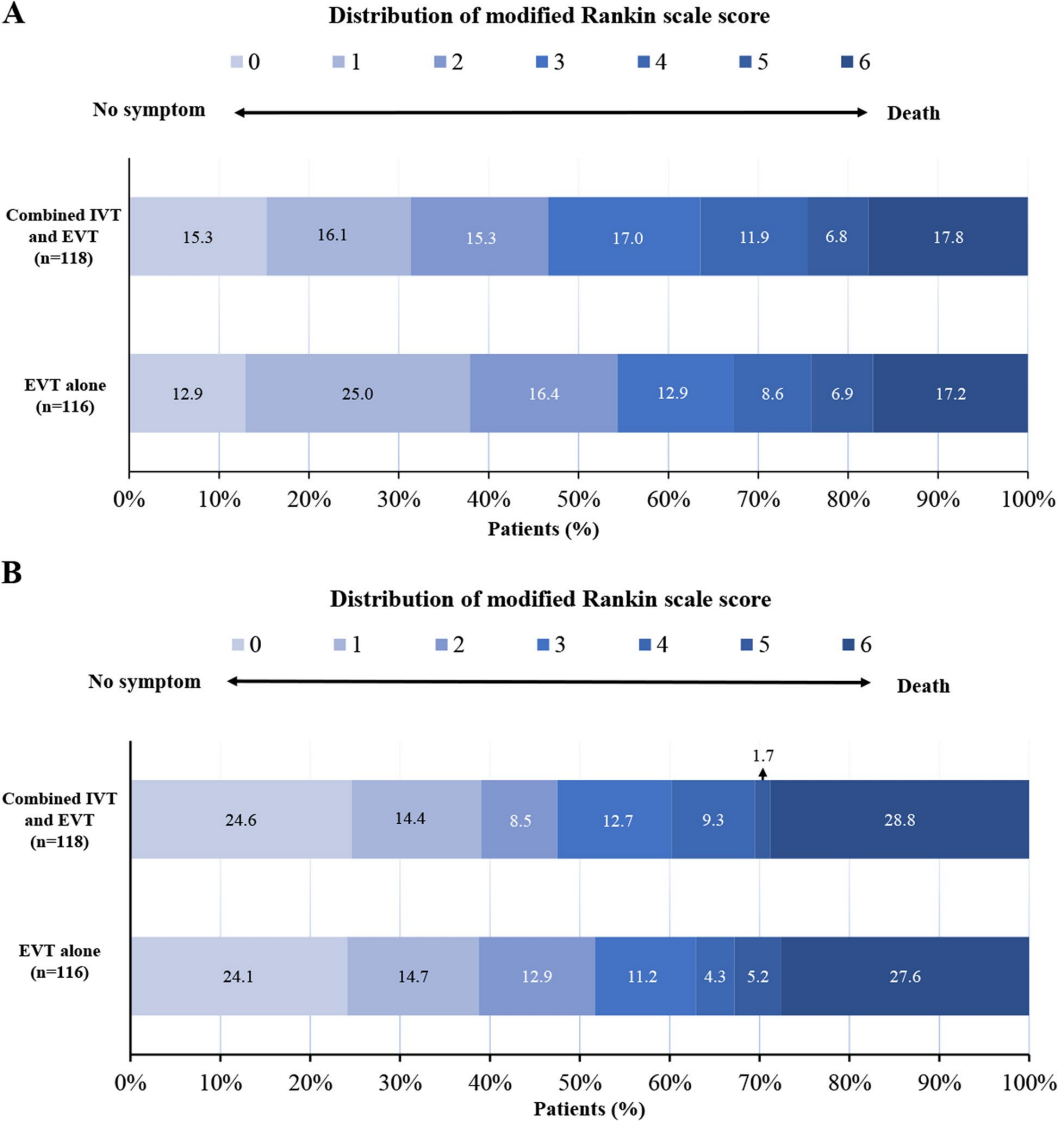
Distribution of the Modified Rankin Scale Score at 3 months and 18 months. Panel **A** shows the distribution of the Modified Rankin Scale Score at 3 months. Panel **B** shows the distribution of the Modified Rankin Scale Score at 18 months

**Fig. 4:**
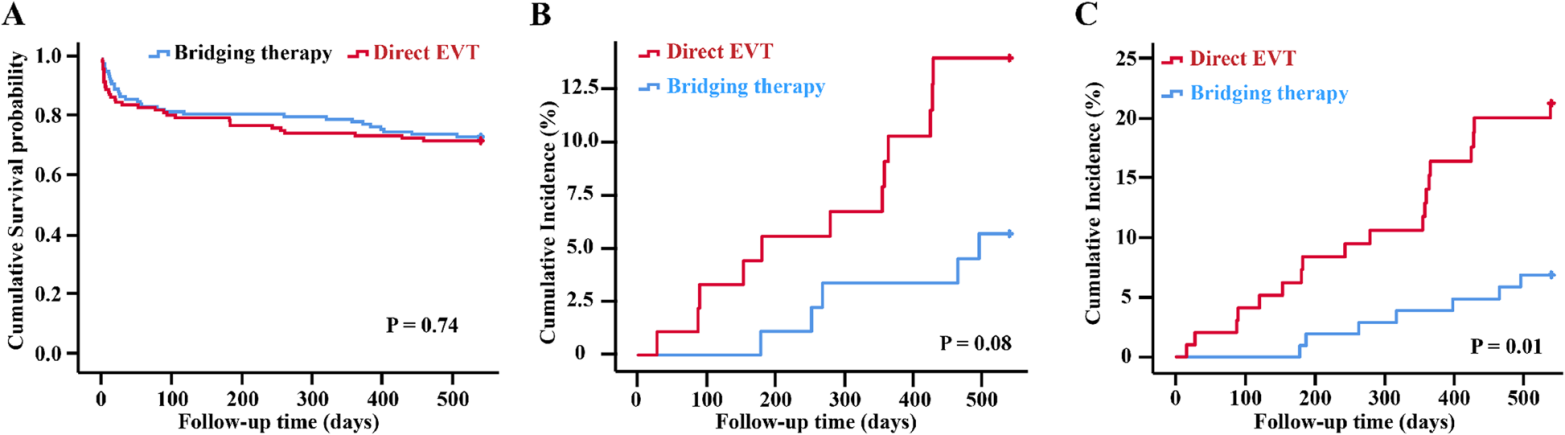
Kaplan-Meier event curves at 18 months. Panel **A** shows the cumulative survival probability in the overall population over a period of 18 months. Panel **B** shows the cumulative incidence of recurrence. Panel **C** shows the cumulative incidence of major vascular event (ischemic stroke, decompensated heart failure, and revascularization surgery for peripheral arterial disease) during the 18-month follow-up period after randomization. The *P* value is calculated with log-Rank test

Compared to 3 months, the mortality was increased to 27.6% in the EVT alone group and 28.8% in the bridging therapy group at follow-up to 18 months (Fig. 3) and was consistent with the primary analysis (eFigure 1). The Kaplan-Meier estimate of the mortality continued to increase during the follow-up term, and no significant difference between the treatment groups was observed (*P* = 0.74) (Fig. 4A).

#### Stroke recurrence

A total of 17 patients with stroke recurrence were reported. Among the stroke recurrence, 12 patients with stroke recurrence in the EVT alone group, and other 5 patients occurred in the bridging therapy group. The probability of stroke recurrence had no significant difference between the treatment groups during the follow-up term with the Kaplan-Meier estimate (*P* = 0.08) (Table 3 and Fig. 4B). The stroke recurrence was consistent with the primary analysis (eTable 3 in Supplement).

**Table 3:** Reported Severe Adverse Events and Procedure Associated Complications

	EVT alone group (*n* = 116)	Bridging therapy group (*n* = 118)
Reported Severe Adverse Events	25 (21.6)	14 (11.9)
Ischemic stroke	12 (10.3)	4 (3.4)
Decompensated heart failure	6 (5.2)	3 (2.5)
Revascularization surgery for PAD	1 (0.9)	0
Other	6 (5.2)	7 (5.9)

*Abbreviations*: *EVT* Endovascular treatment, *IVT* Intravenous thrombolysis, *PAD* Peripheral arterial disease

#### Major vascular events

In the extend follow-up period, 39 patients with major vascular events were reported. Among the major vascular events, 25 of the events occurred during 116 person-years of follow-up in the EVT alone group, and other 14 events occurred during 115 person-years of follow-up in the bridging therapy group. The probability of major vascular events in EVT alone group had significantly increased compared to the bridging therapy group during the follow-up term with the Kaplan-Meier estimate (*P* = 0.01) (Table 3 and Fig. 4C). The major vascular events were consistent with the primary analysis (eTable 3 in Supplement).

#### Sensitivity analysis

The pooled effect on the primary outcome after multiple imputation (adjusted odds ratio [OR], 1.21 [95% CI, 0.68–2.15]) and secondary outcomes (i.e., mRS 0–1, adjusted OR, 0.96 [95% CI, 0.53–1.74]) (Table 2) were similar to the results of the main analysis of the primary and secondary outcomes without imputation (eTable 2 in Supplement).

#### Subgroup analyses

After adjustment for age, baseline NIHSS score, baseline ASPECTS, occlusion site, and time from onset to randomization, multivariate logistic regression analysis revealed that no significant interactions (effect modifications) were observed between the prespecified subgroups (eFigure 2 in Supplement), which were defined according to baseline characteristics, and treatment at 18 months. The treatment effect remained consistent in all prespecified subgroups (eFigure 3 in Supplement). However, some subgroups were small, which resulted in wide confidence intervals.

## Discussion

The results of the extended follow-up evaluation of the DEVT trial showed that EVT alone was noninferior to intravenous alteplase combined with EVT regarding to functional outcome at 18 months, which was similar to the originally reported results at 90 days. The between-group difference of the percentage of patients who were functionally independent (i.e., a mRS score of 0 to 2) at 18 months (4.3%) was lower than the results at 90 days (7.7%), and the lower boundary of the CI (− 8.4%) was greater than the prespecified noninferiority margin of − 10%.

The percentage of patients with independent functional outcome at 18 months was lower than the percentages at 90 days in the EVT alone group, although this difference was not statistically significant, whereas in the combined treatment group, the percentage was slightly higher at 18 months than the percentage at 90 days. This may have contributed to the differences across the original trial and extended follow-up study. A possible explanation for the lower rate of functional independence at 18 months in the EVT alone group included the higher proportion of both stroke recurrence (10.3% vs 3.4%) and decompensated heart failure (5.2% vs 2.5%) over the combined treatment group during the extended follow-up period, because both these factors appeared to be associated with worse functional outcomes after endovascular treatment in long-term studies [18].

In addition to the above results, notable differences were observed between the two time points. First, the mortality rate at 18 months was higher than the risk of death at 90 days in both groups. Second, the percentage of patients with excellent outcome at 18 months was higher than the percentages at 90 days in the combined treatment group, although this difference was not statistically significant. Third, the utility index of EQ-5D-5L was slightly lower at 18 months in the EVT alone group but did not differ substantially between the two time points.

We observed more major vascular events in the endovascular treatment alone group but did not differ substantially between the two groups during the extended follow-up period. This observation may be attributed to a higher proportion of patients with diabetes, coronary heart disease and large artery disease co-morbidity in the EVT alone group than in the combined treatment group, although there was no significant difference. A recent meta-analysis showed that predictors of long-term stroke recurrences were both modifiable and nonmodifiable. Increasing age, male sex, diabetes mellitus, smoking, cardiac disease, and stroke etiology (large artery disease) were all associated with increased risk of late recurrences. Moreover, many of the nonmodifiable and modifiable risk factors which contribute to stroke recurrences were also important predictors of cardiovascular events.

Acute stroke studies typically reported the primary outcome measure at 90 days. Longer follow-up assessments were prone to result in patients being lost to follow-up, which carried a substantial risk of bias. In that regard, our study was unique in that data for the primary outcome were available for all enrolled patients at all follow-up times, with the exception of three patients at 18 months, conferring strong internal validity to our findings.

### Limitations

Our trial has several limitations. The noninferiority margin used in the original trial was not selected using the minimal clinically important difference or fixed margin methods. The noninferior margin of 10% is broad, reaching no consensus in the clinical community, which may lead to concerns about the robustness of study results. Furthermore, the early termination and small sample size of the trial may create potential limitation affecting our findings that the treatment effect observed with endovascular treatment alone at 3 months was sustained at 18 months. Finally, missing data of long-term outcome at 18 months may introduce a substantial risk of bias. However, a sensitivity analysis in which missing outcomes were imputed by means of model-based imputation showed results that were similar to those of the main analysis of the primary outcome, which suggests a limited effect of bias.

## Conclusion

Among patients with ischemic stroke due to proximal anterior circulation occlusion within 4.5 hours from onset, endovascular treatment alone, compared with IV alteplase plus endovascular treatment, might demonstrate noninferiority regarding favorable functional outcome at 18 months. However, these findings should be interpreted with respect to the clinical acceptability of the noninferiority margin.

## Supplementary Information

**Additional file 1: eTable 1.** Baseline characteristics and workflow measures of per-protocol analysis. **eTable 2.** Modified Rankin Scale Score at 90 days and secondary outcomes of per-protocol analysis. **eTable 3.** Reported severe adverse events and procedure associated complications of per-protocol analysis. **eFigure 1.** Distribution of the Modified Rankin Scale Score at 90 days of per-protocol analysis. **eFigure 2.** Analysis of functional independence at 18 months in prespecified subgroups. **eFigure 3.** Analysis of functional independence at 18 months in prespecified subgroups of per-protocol analysis.

## Data Availability

Data are available from the corresponding author upon reasonable requests.
