# Supplementary material for: Effect of direct endovascular treatment versus standard bridging therapy in large artery anterior circulation stroke (DEVT): 18-month follow-up of a randomized controlled trial
Source: BMC Neurol. 2023 Feb 27;23:84. doi: 10.1186/s12883-023-03111-y (PMC9969708; doi:10.1186/s12883-023-03111-y)
Supplement: Supplementary file 1 — Additional file 1: eTable 1. Baseline characteristics and workflow measures of per-protocol analysis. eTable 2. Modified Rankin Scale Score at 90 days and secondary outcomes of per-protocol analysis. eTable 3. Reported severe adverse events and procedure associated complications of per-protocol analysis. eFigure 1. Distribution of the Modified Rankin Scale Score at 90 days of per-protocol analysis. eFigure 2. Analysis of functional independence at 18 months in prespecified subgroups. eFigure 3. Analysis of functional independence at 18 months in prespecified subgroups of per-protocol analysis. [file 12883_2023_3111_MOESM1_ESM.docx]

**Effect of Direct Endovascular Treatment Versus Standard Bridging Therapy in Large Artery Anterior Circulation Stroke (DEVT): 18-month follow-up of a randomized controlled trial**

**Supplemental Material**

**Content**

**eTable 1.** Baseline characteristics and workflow measures of per-protocol analysis

**eTable 2.** Modified Rankin Scale Score at 90 days and secondary outcomes of per-protocol analysis

**eTable 3.** Reported severe adverse events and procedure associated complications

**eFigure 1.** Distribution of the Modified Rankin Scale Score at 90 days of per-protocol analysis

**eFigure 2.** Analysis of functional independence at 18 months in prespecified subgroups

**eFigure 3.** Analysis of functional independence at 18 months in prespecified subgroups of per-protocol analysis

eTable 1. Baseline Characteristics and Workflow Measures

|  | EVT alone group (n = 116) | Bridging therapy group (n = 115) |
| --- | --- | --- |
| **Demographic characteristics** |  |  |
| Age,median (IQR), y | 70 (60–77) | 70 (59–78) |
| Male sex no.% | 66 (56.9) | 64 (55.7) |
| Medical history |  |  |
| Hypertension | 69 (59.5) | 72 (62.6) |
| Atrial fibrillation | 62 (53.4) | 59 (51.3) |
| Smoking | 28 (24.1) | 29 (25.2) |
| Diabetes | 25 (21.6) | 20 (17.4) |
| Hyperlipidemia | 18 (15.5) | 22 (19.1) |
| Coronary heart disease | 30 (25.9) | 17 (14.8) |
| Clinical characteristics |  |  |
| Prestroke mRS score |  |  |
| 0 | 110 (94.8) | 105 (91.3) |
| 1 | 6 (5.2) | 10 (8.7) |
| Stroke etiology |  |  |
| Cardioembolism | 65 (56.0) | 67 (58.3) |
| Large artery atherosclerosis | 32 (27.6) | 28 (24.3) |
| Unknown | 15 (12.9) | 19 (16.5) |
| Other | 4 (3.4) | 1 (0.9) |
| **Imaging characteristics** |  |  |
| Baseline ASPECTS <8 | 50 (43.1) | 51 (44.3) |
| Location of intracranial occlusion |  |  |
| Intracranial internal carotid artery | 18 (15.5) | 16 (13.9) |
| M1 middle cerebra lartery segment | 95 (81.9) | 97 (84.3) |
| M2 middle cerebral artery segment | 3 (2.6) | 2 (1.7) |
| Baseline NIHSS score ≥16 | 59 (50.9) | 62 (53.9) |
| **Workflow times** |  |  |
| Time from stroke onset to randomization |  |  |
| 0-3h | 68 (58.6) | 66 (57.4) |
| 3-6h | 48 (41.4) | 49 (42.6) |
| Arrival to intravenous alteplase, median (IQR), min | NA | 62 (49–81) |
| Arrival to arterial puncture, median (IQR), min | 101 (80–135) | 104 (80–132) |
| Onset to puncture, median (IQR), min | 200 (155–247) | 206 (177–255) |

Abbreviations: ASPECTS, Alberta Stroke Program Early Computed Tomography Score; IQR, interquartile range; EVT, endovascular treatment; IVT, intravenous thrombolysis; NIHSS, National Institutes of Health Stroke Scale; NA, not applicable.

eTable 2. Modified Rankin Scale Score at 90 Days and Secondary Outcomes

Abbreviations: mRS, modified Rankin Scale Score; EQ-5D-5L, European Quality of Life 5-Dimensions 5-Level questionnaire; IQR, interquartile range; EVT, endovascular treatment; IVT, intravenous thrombolysis; OR, odds ratio; CI, confidence interval.

|  | EVT alone group (n =116) | Bridging therapy group (n = 115) | Unadjusted difference (95% CI) | Unadjusted OR  (95% CI) | Adjusted OR  (95% CI) |
| --- | --- | --- | --- | --- | --- |
| Primary efficacy outcome |  |  |  |  |  |
| Functional independence | 60 (51.7%) | 56 (48.7%) | 3.0 (-9.7–15.6) | 1.13 (0.67–1.89) | 1.17 (0.66–2.09) |
| Secondary efficacy outcome |  |  |  |  |  |
| Excellent outcome | 45 (38.8%) | 46 (40.0%) | 1.2 (-11.2–13.6) | 0.95 (0.56–1.61) | 0.94 (0.52–1.71) |
| Disability level, median (IQR), mRS score | 2 (1–6) | 3 (0–6) | 0 (0–0) | 1.01 (0.64–1.60) | 1.00 (0.62–1.61) |
| EQ-5D-5L score, median (IQR) | 0.81 (0.00–1.00) | 0.78 (0.00–1.00) | 0 (0–0.005) | -0.006 (-0.12–0.11) | -0.003 (-0.11–0.10) |

|  | EVT alone group (n =116) | Bridging therapy group (n = 118) |
| --- | --- | --- |
| Reported Severe Adverse Events | 25 (26.0) | 14 (14.4) |
| Ischemic stroke | 12 (12.5) | 4 (4.3) |
| Decompensated heart failure | 6 (6.3) | 3 (3.2) |
| Revascularization surgery for PAD | 1 (1.0) | 0 |
| Other | 6 (6.3) | 7 (7.4) |
| Recurrence | 12 (13.0) | 4 (4.5) |

eTable 3. Reported Severe Adverse Events and Procedure Associated Complications

Abbreviations: EVT, endovascular treatment; IVT, intravenous thrombolysis; PAD, peripheral arterial disease.


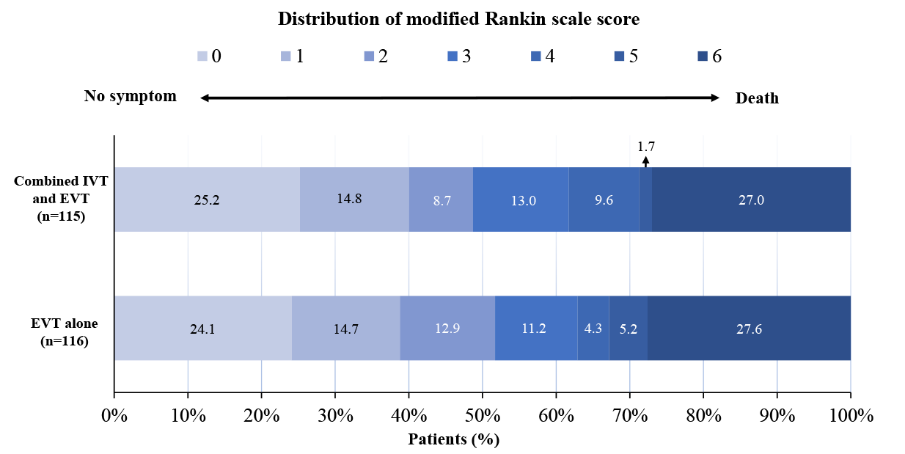
**eFigure 1.** Distribution of the Modified Rankin Scale Score at 90 days of per-protocol analysis

**eFigure 2.** Analysis of functional independence at 18 months in prespecified subgroups


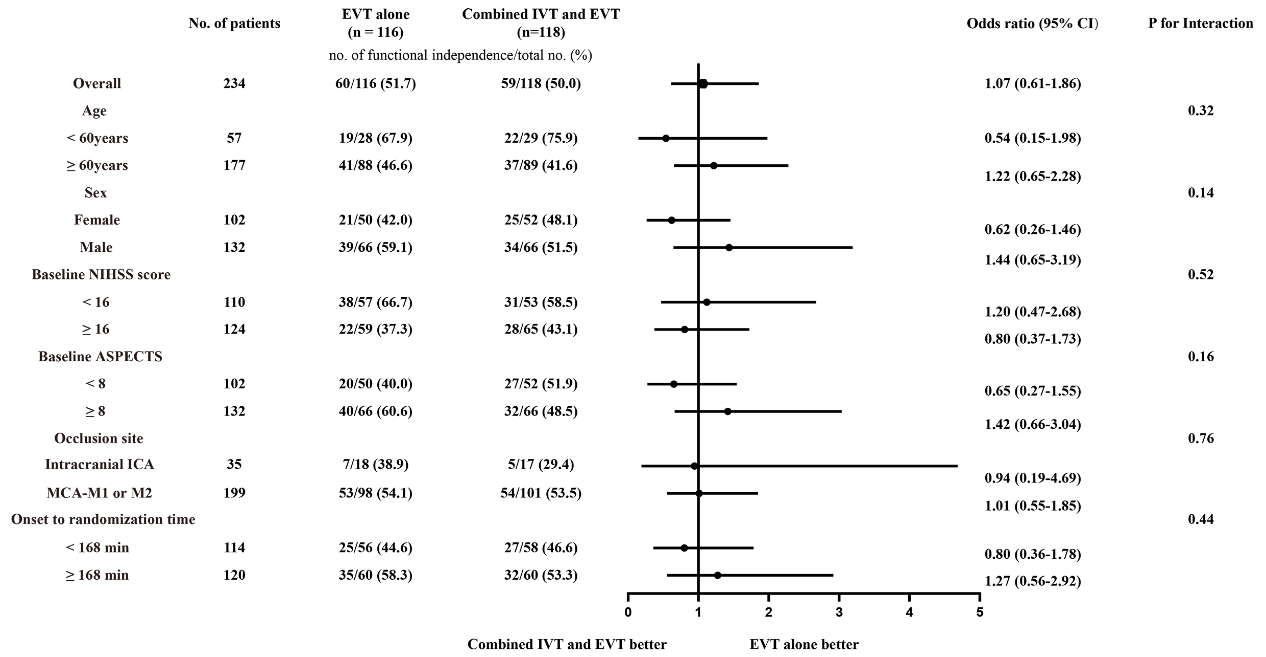


This forest plot shows that there was no evidence of heterogeneity of treatment effect across in most prespecified subgroups. The odds ratio was calculated by using logistic regression taking the following variables into account: age, baseline NIHSS score, baseline ASPECTS, occlusion site, and time from onset to randomization. NIHSS National Institutes of Health Stroke Scale; ASPECTS Alberta Stroke Program Early CT Score; CE denotes cardioembolism, CI confidence interval, ICA internal carotid artery, LAA large artery atherosclerosis, MCA-M1 or M2 the first or second segment of middle cerebral artery.

**eFigure 3.** Analysis of functional independence at 18 months in prespecified subgroups of per-protocol analysis


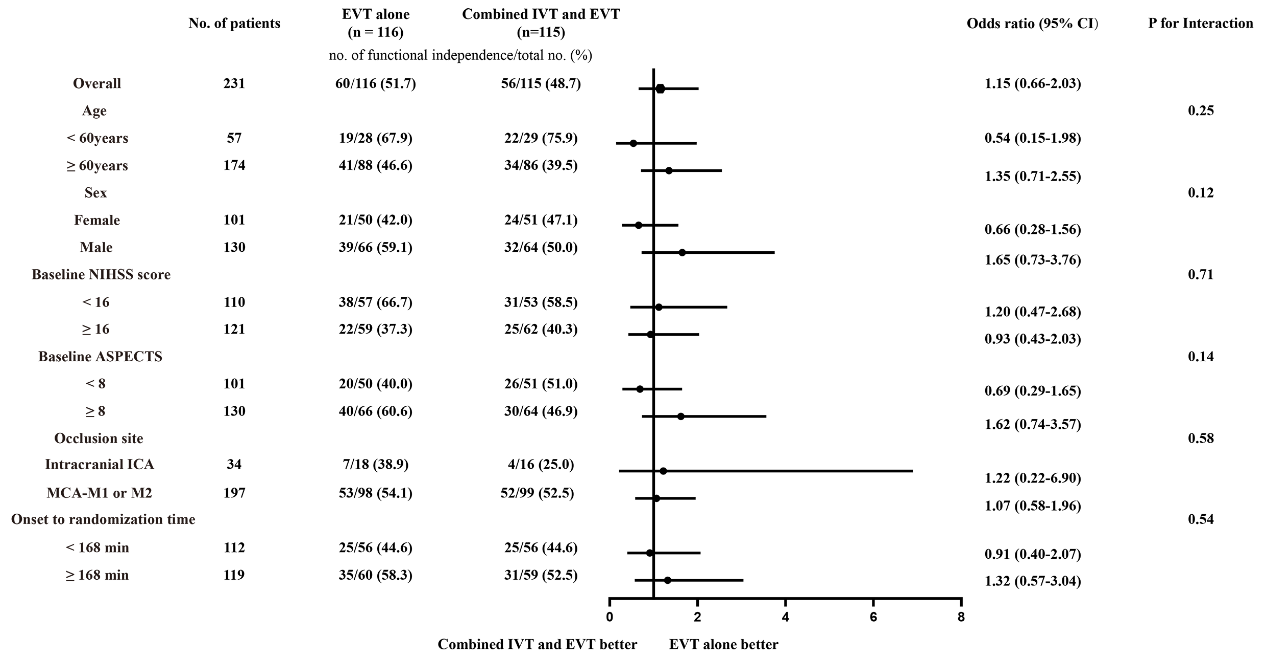


This forest plot shows that there was no evidence of heterogeneity of treatment effect across in most prespecified subgroups. The odds ratio was calculated by using logistic regression taking the following variables into account: age, baseline NIHSS score, baseline ASPECTS, occlusion site, and time from onset to randomization. NIHSS National Institutes of Health Stroke Scale; ASPECTS Alberta Stroke Program Early CT Score; CE denotes cardioembolism, CI confidence interval, ICA internal carotid artery, LAA large artery atherosclerosis, MCA-M1 or M2 the first or second segment of middle cerebral artery.
